# Supplementary material for: Association of Hospital Characteristics and Previous Hospitalization-Related Experiences with Patients’ Perceptions of Hospital Care in China
Source: Int J Environ Res Public Health. 2022 Jun 27;19(13):7856. doi: 10.3390/ijerph19137856 (PMC9265902; doi:10.3390/ijerph19137856)
Supplement: Supplementary file 1 [file ijerph-19-07856-s001.zip › ijerph-1677652-supplementary.pdf]

Supplementary Online Content

Association of Hospital Characteristics and Previous Hospitalization-related Experiences with Patients' Perception of Hospital Care in China

Figure S1. Flowchart for recruitment and response rates of the participants.

Table S1. Correlations among index of patients' perception of hospital care

Table S2. Patients' Perception of Hospital Care

Table S3. Difference of Patients' perception of hospital care in detail according to regional characteristics

Table S4. Patients' perception of hospital care according to hospital characteristics and patients' previous hospitalization-related experiences-Results of all independent variables

Table S5. Difference of Patients' perception of hospital care in detail according to regional characteristics

Table S6. Difference of three dimensions' and total score of patients' perception of hospital care according to regional characteristics

This supplementary material has been provided by the authors to give readers additional information about their work.

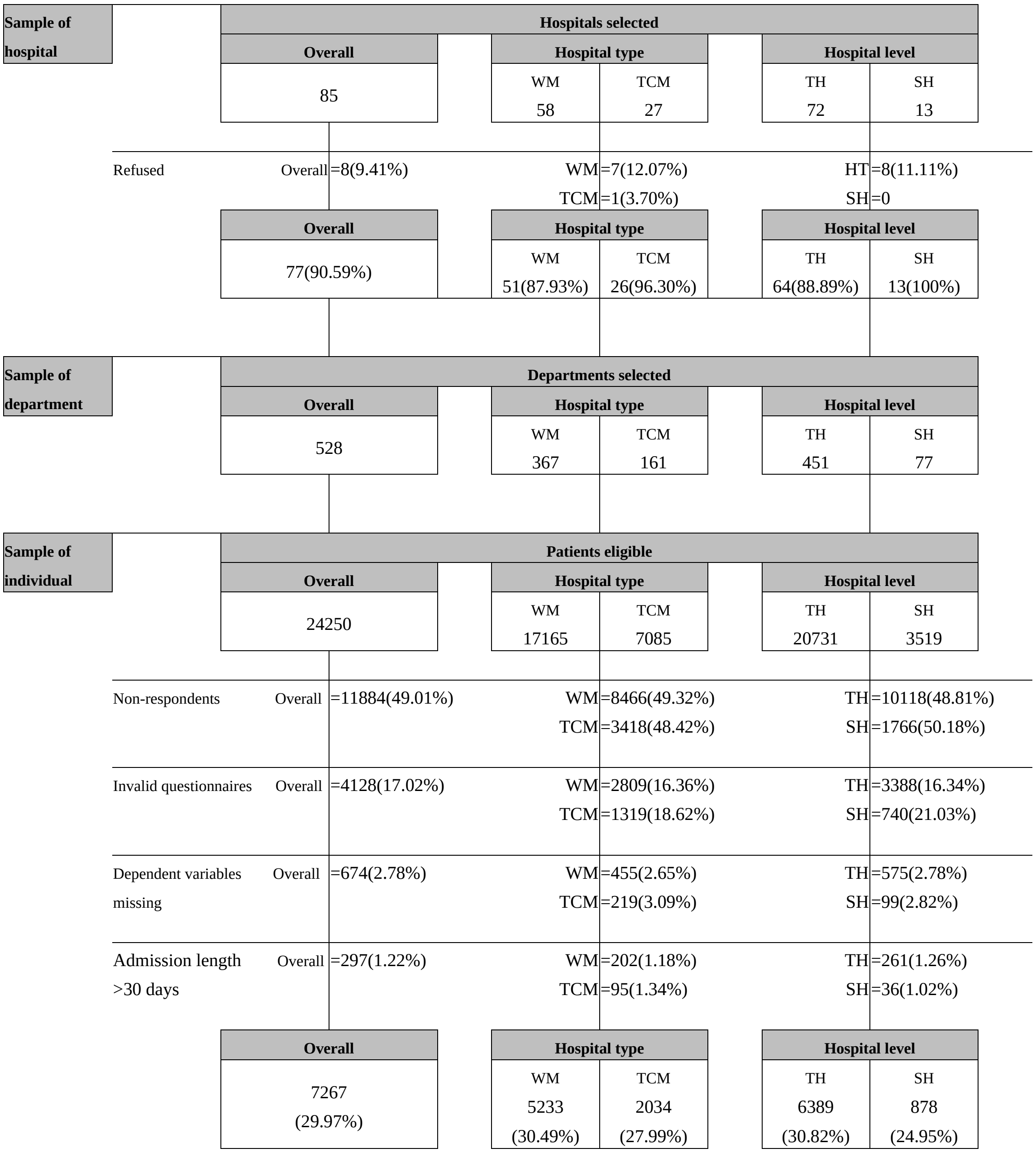

Figure S1. Flowchart for recruitment and response rates of the participants. WM, Western Medicine. TCM, Traditional Chinese Medicine. TH, Tertiary Hospital. SH, Secondary Hospital.

Table S1. Patients' Perception of Hospital Care

	Very poor	Poor	Neither poor nor good	Good	Very good
**Patient care provided by doctors**					
Communication with doctors	4.22	8.50	19.90	28.32	39.06
Accessibility to doctors	1.80	5.96	24.52	36.20	31.51
Doctors' concern for patients' mood	2.52	5.46	28.55	36.04	27.43
Involvement in medical services	3.19	5.66	24.17	42.06	24.92
**Patient care provided by nurses**					
Communication with nurses	5.36	6.90	15.74	26.25	45.75
Accessibility to nurses	0.55	1.56	12.90	36.74	48.25
Nurses' concern for patients' mood	2.75	4.15	28.15	35.02	29.93
Involvement in nursing services	3.45	4.32	23.63	40.41	28.20
**Patient care provided by hospital organization**					
Clean environment	0.70	2.17	26.91	42.34	27.87
Quiet environment	1.53	6.06	33.51	38.11	20.79
Convenience of medical examination	1.70	4.50	36.15	39.40	18.26

Table S2. Correlations among index of patients' perception of hospital care

	1	2	3	4	5	6	7	8	9	10	11
1.Communication with doctors	1	0.234**	0.171**	0.156**	0.172**	0.581**	0.098**	0.117**	0.169**	0.122**	0.149**
2.Accessibility to doctors		1	0.647**	0.520**	0.473**	0.161**	0.426**	0.430**	0.385**	0.354**	0.420**
3.Doctors' concern for patients' mood			1	0.570**	0.406**	0.110**	0.571**	0.517**	0.402**	0.377**	0.419**
4.Involvement in medical services				1	0.373**	0.117**	0.428**	0.520**	0.347**	0.310**	0.345**
5.Accessibility to nurses					1	0.205**	0.553**	0.525**	0.439**	0.343**	0.374**
6.Communication with nurses						1	0.065**	0.092**	0.163**	0.081**	0.132**
7.Nurses' concern for patients' mood							1	0.637**	0.410**	0.364**	0.380**
8.Involvement in nursing services								1	0.416**	0.358**	0.364**
9.Clean environment									1	0.634**	0.487**
10.Quiet environment										1	0.421**
11.Convenience of medical exam											1

Table S3. High ratings on individual components of patients' perception of hospital care according to hospital characteristics and patients' previous hospitalization-related experiences

Variables	Provided by doctors								Provided by nurses								Provided by hospital organization					
	Communication with doctors		Accessibility to doctors		Doctors' concern for patients' mood		Involvement in medical services		Communication with nurses		Accessibility to nurses		Nurses' concern for patients' mood		Involvement in nursing services		Quiet environment		Clean environment		Convenience of medical examn	
	%	*p*	%	*p*	%	*p*	%	*p*	%	*p*	%	*p*	%	*p*	%	*p*	%	*p*	%	*p*	%	*p*
**Hospital characteristics**																						
Hospital technical level																						
Secondary hospital	33.61	0.001	31.70	0.813	22.81	<0.001	20.58	0.005	40.82	0.004	45.26	0.021	27.49	0.030	25.50	<0.001	23.07	<0.001	19.77	0.204	17.45	0.386
Tertiary hospital	39.78		31.50		28.05		25.50		46.40		48.66		30.26		28.57		28.50		20.91		18.36	
Hospital type																						
Western medicine	39.26	0.406	31.08	0.182	27.37	0.163	24.87	0.504	46.34	0.371	47.87	0.706	30.15	0.869	28.14	0.178	28.70	<0.001	20.76	0.232	18.43	0.005
Traditional Chinese medicine	38.56		32.65		27.60		25.04		44.20		49.24		29.37		28.34		25.74		20.83		17.78	
Academic status																						
Nonteaching	39.58	0.110	32.82	<0.001	27.91	0.168	25.55	0.177	45.93	0.443	47.76	0.311	29.71	0.105	28.37	0.093	27.32	0.097	21.19	0.019	18.25	0.320
Teaching	37.25		26.94		25.70		22.69		45.15		50.00		30.71		27.56		29.81		19.38		18.26	
Ratio of doctors to ward beds																						
<0.20	40.12	0.109	33.04	0.613	28.66	0.219	25.81	0.174									26.85	0.176	20.14	0.043	19.82	0.016
0.20-0.30	38.31		30.68		27.36		25.02										27.32		20.23		17.26	
≥0.30	38.95		30.98		25.89		23.57										30.12		22.60		17.91	
Ratio of nurses to ward beds																						
<0.4									45.92	0.100	48.42	0.090	30.53	0.125	28.99	0.140	27.70	0.763	21.56	0.074	18.73	0.292
0.4-0.6									45.01		47.29		28.54		26.95		27.91		20.07		17.78	
≥0.6									48.48		51.99		32.24		27.40		29.21		17.33		16.39	
**Previous hospitalization-related experiences**																						
Current admission length (days)																						
1-3	39.42	0.065	31.48	0.127	26.32	0.001	23.81	<0.001	46.17	0.066	48.64	0.179	29.17	0.038	26.98	0.004	29.38	0.355	21.56	0.089	18.31	0.014
4-7	38.91		30.11		24.33		23.39		46.00		47.00		27.64		25.99		27.21		19.80		16.60	
8-14	39.92		32.10		29.91		24.88		46.14		49.73		31.93		29.72		27.19		20.46		18.30	
15-21	36.47		34.90		30.30		29.87		44.81		50.78		32.86		33.14		30.73		23.90		21.19	
≥22days	41.00		33.33		33.92		30.68		46.90		51.62		33.63		32.74		28.32		21.18		21.53	
Number of previous admissions in the last three years																						
0	43.62	<0.001	32.03	<0.001	27.52	<0.001	24.79	<0.001	49.94	<0.001	48.81	0.004	29.32	0.004	27.22	<0.001	29.29	0.006	21.70	0.338	19.32	<0.001
1	31.11		26.46		22.92		21.79		39.03		45.38		29.16		27.13		24.84		18.40		15.26	
2	30.86		30.38		27.54		25.12		38.16		46.53		30.62		29.63		26.08		20.22		18.66	
3	35.94		33.85		29.43		24.42		42.45		50.65		31.43		30.99		27.27		20.83		15.06	
≥4 次	43.84		40.69		36.41		32.79		48.64		53.80		34.96		34.42		30.14		22.10		19.75	
Hospital selection by personal recommendations																						
No	40.17	0.252	31.30	0.005	26.13	<0.001	24.35	0.019	47.32	0.016	47.54	0.094	28.08	<0.001	26.90	0.004	26.84	0.004	20.31	0.013	18.00	0.264
Yes	37.70		31.61		28.70		25.57		44.05		49.21		32.03		29.65		29.20		21.29		18.47	
Hospital selection by advertisements																						
No	40.01	<0.001	31.34	0.015	27.01	0.173	24.41	<0.001	46.90	<0.001	48.43	<0.001	28.98	<0.001	27.42	0.006	27.45	<0.001	20.28	0.044	17.95	0.719
Yes	31.60		30.57		28.20		26.51		37.56		46.80		36.45		32.94		30.89		24.02		19.55	

Table S4. Multivariate logistic regression model to examine the association of hospital characteristics and patients' previous hospitalization-related experiences with patients' perception of hospital care

Variables	Provided by doctors				Provided by nurses				Provided by hospital organization		
	**Communication with doctors**	**Accessibility to doctors**	**Doctors' concern for patients' mood**	**Involvement in medical services**	**Communication with nurses**	**Accessibility to nurses**	**Nurses' concern for patients' mood**	**Involvement in nursing services**	**Quiet environment**	**Clean environment**	**Convenience of medical exam**
	**OR(95%CI)**	**OR(95%CI)**	**OR(95%CI)**	**OR(95%CI)**	**OR(95%CI)**	**OR(95%CI)**	**OR(95%CI)**	**OR(95%CI)**	**OR(95%CI)**	**OR(95%CI)**	**OR(95%CI)**
**Hospital characteristics**											
Hospital technical level											
Secondary hospital	1.00	1.00	1.00	1.00	1.00	1.00	1.00	1.00	1.00	1.00	1.00
Tertiary hospital	1.26(1.06,1.49) **	0.92(0.77,1.09)	1.24(1.02,1.5) *	1.28(1.06,1.56) *	1.27(1.08,1.5) **	1.16(0.99,1.36)	1.09(0.91,1.31)	1.15(0.95,1.38)	1.35(1.12,1.63) **	1.07(0.88,1.31)	0.99(0.8,1.23)
Hospital type											
Western medicine	1.00	1.00	1.00	1.00	1.00	1.00	1.00	1.00	1.00	1.00	1.00
Traditional Chinese medicine	0.99(0.88,1.12)	1.15(1.01,1.3) *	0.97(0.85,1.11)	0.99(0.87,1.13)	0.9(0.8,1.01)	1.04(0.93,1.16)	0.93(0.82,1.06)	0.98(0.86,1.11)	0.8(0.7,0.91) ***	0.95(0.82,1.09)	0.91(0.78,1.06)
Academic status											
Nonteaching	1.00	1.00	1.00	1.00	1.00	1.00	1.00	1.00	1.00	1.00	1.00
Teaching	0.86(0.75,0.98) *	0.76(0.66,0.88) ***	0.85(0.74,0.99) *	0.83(0.71,0.96) *	0.91(0.8,1.04)	1.08(0.95,1.22)	1.06(0.92,1.22)	0.93(0.81,1.07)	1.11(0.96,1.27)	0.91(0.77,1.06)	1.05(0.88,1.24)
Ratio of doctors to ward beds											
<0.20	1.00	1.00	1.00	1.00					1.00	1.00	1.00
0.20-0.30	0.88(0.78,0.99) *	0.92(0.81,1.05)	0.91(0.8,1.04)	0.94(0.82,1.08)					1(0.87,1.14)	1.08(0.93,1.25)	0.81(0.69,0.95) **
≥0.30	0.96(0.84,1.1)	0.95(0.82,1.1)	0.9(0.77,1.06)	0.94(0.8,1.11)					1.16(0.99,1.36)	1.33(1.12,1.59) **	0.9(0.74,1.08)
Ratio of nurses to ward beds											
<0.4					1.00	1.00	1.00	1.00	1.00	1.00	1.00
0.4-0.6					0.98(0.88,1.1)	0.95(0.85,1.06)	0.91(0.81,1.03)	0.93(0.82,1.05)	0.95(0.84,1.08)	0.88(0.76,1.01)	0.96(0.82,1.11)
≥0.6					1.11(0.89,1.38)	1.18(0.95,1.47)	1.16(0.91,1.47)	0.99(0.77,1.26)	1.03(0.8,1.32)	0.7(0.52,0.94) *	0.88(0.64,1.2)
**Previous hospitalization-related experiences**											
Current admission length (days)											
1-3	1.00	1.00	1.00	1.00	1.00	1.00	1.00	1.00	1.00	1.00	1.00
4-7	1(0.87,1.15)	0.92(0.79,1.07)	0.92(0.78,1.08)	0.94(0.8,1.11)	0.99(0.86,1.13)	0.95(0.83,1.1)	0.93(0.8,1.09)	0.96(0.82,1.12)	0.93(0.8,1.08)	0.92(0.78,1.09)	0.91(0.76,1.1)
8-14	1.05(0.91,1.22)	0.98(0.83,1.14)	1.19(1.01,1.4) *	1.03(0.87,1.22)	1.04(0.9,1.2)	1.05(0.91,1.21)	1.14(0.97,1.33)	1.17(1,1.38)	0.92(0.79,1.08)	0.98(0.82,1.17)	0.99(0.82,1.2)
15-21	0.93(0.76,1.14)	1.15(0.94,1.41)	1.25(1.01,1.56) *	1.3(1.04,1.61) *	0.99(0.81,1.2)	1.06(0.87,1.28)	1.15(0.94,1.42)	1.34(1.09,1.65) **	1.09(0.88,1.35)	1.2(0.95,1.5)	1.15(0.9,1.46)
≥22days	1.13(0.88,1.47)	1.21(0.92,1.58)	1.6(1.22,2.1) ***	1.5(1.13,1.98) **	1.17(0.91,1.51)	1.28(0.99,1.64)	1.35(1.03,1.76) *	1.5(1.14,1.96) **	1.1(0.83,1.44)	1.12(0.83,1.51)	1.36(1,1.85) *
Number of previous admissions in the last three years											
0	1.00	1.00	1.00	1.00	1.00	1.00	1.00	1.00	1.00	1.00	1.00
1	0.59(0.51,0.67) ***	0.7(0.6,0.81) ***	0.74(0.64,0.87) ***	0.83(0.71,0.97) *	0.68(0.6,0.78) ***	0.89(0.78,1.01)	0.93(0.8,1.07)	0.94(0.81,1.09)	0.77(0.66,0.89)***	0.78(0.66,0.92) **	0.7(0.58,0.83) ***
2	0.62(0.52,0.74) ***	0.86(0.72,1.03)	0.93(0.77,1.12)	1.02(0.85,1.24)	0.68(0.58,0.8) ***	0.94(0.8,1.11)	0.98(0.82,1.18)	1.08(0.9,1.29)	0.85(0.71,1.03)	0.9(0.73,1.1)	0.94(0.76,1.16)
3	0.77(0.6,0.97) *	0.93(0.73,1.19)	0.91(0.7,1.18)	0.89(0.68,1.18)	0.81(0.64,1.02)	1.04(0.83,1.31)	0.97(0.75,1.24)	1.15(0.89,1.47)	0.85(0.66,1.1)	0.85(0.64,1.13)	0.66(0.48,0.91) *
≥4times	1.02(0.84,1.24)	1.29(1.05,1.59) *	1.38(1.12,1.71) **	1.51(1.22,1.87) ***	1.02(0.84,1.24)	1.25(1.03,1.52) *	1.32(1.07,1.62) **	1.42(1.15,1.75) **	1.05(0.85,1.3)	1.04(0.82,1.32)	0.98(0.76,1.25)
Hospital selection by personal recommendations											
No	1.00	1.00	1.00	1.00	1.00	1.00	1.00	1.00	1.00	1.00	1.00
Yes	0.94(0.84,1.04)	1.07(0.96,1.2)	1.21(1.07,1.36) **	1.1(0.97,1.24)	0.91(0.82,1.01)	1.07(0.96,1.18)	1.16(1.04,1.3) **	1.14(1.02,1.28) *	1.09(0.97,1.22)	1.05(0.92,1.19)	1.02(0.9,1.17)
Hospital selection by advertisements											

No	1.00	1.00	1.00	1.00	1.00	1.00	1.00	1.00	1.00	1.00	1.00
Yes	0.75(0.64,0.89) **	0.94(0.78,1.12)	1.01(0.84,1.21)	1.1(0.92,1.32)	0.78(0.67,0.92) **	0.9(0.77,1.06)	1.3(1.1,1.54) **	1.22(1.03,1.45) *	1.12(0.94,1.33)	1.19(0.99,1.44)	1.12(0.92,1.38)

Note:***: $p < 0.001$;**: $p < 0.01$;*: $p < 0.05$; All models were adjusted for the following patient sociodemographic characteristics: sex, age, educational level, marital status, medical insurance, and self-reported economic status.

Table S5. Difference of total and three dimensions' score of patients' perception of hospital care according to regional characteristics

	%	**Provided by doctors**		**Provided by nurses**		**Provided by hospital organization**		**Total**	
		**Mean score (95%CI)**	***P* value**	**Mean score (95%CI)**	***P* value**	**Mean score (95%CI)**	***P* value**	**Mean score (95%CI)**	***P* value**
North(Beijing)	9.96	69.17(67.85,70.48)	<0.001	75.41(74.16,76.65)	<0.001	69.66(68.33,71)	<0.001	71.41(70.34,72.48)	<0.001
South(Guangdong)	17.70	68.91(67.91,69.9)		74.02(73.1,74.94)		68.52(67.64,69.4)		70.48(69.7,71.26)	
East(Jiangsu, Shandong)	30.18	73.71(72.92,74.5)		77.45(76.71,78.18)		71.74(70.98,72.5)		74.3(73.64,74.95)	
West(Gansu, Yunnan)	25.59	72.54(71.72,73.36)		74.51(73.72,75.3)		69.53(68.68,70.37)		72.19(71.51,72.88)	
Central(Hubei)	16.57	68.31(67.28,69.35)		72.69(71.73,73.65)		65.87(64.86,66.88)		68.96(68.13,69.79)	

Table S6. Difference of patients’ perception of hospital care in detail according to regional characteristics.

Variables	Provided by doctors								Provided by nurses								Provided by hospital organization					
	Communication with doctors		Accessibility to doctors		Doctors' concern for patients’ mood		Involvement in medical services		Communication with nurses		Accessibility to nurses		Nurses' concern for patients’ mood		Involvement in nursing services		Clean environment		Quiet environment		Convenience of medical examn	
	%	*p*	%	*p*	%	*p*	%	*p*	%	*p*	%	*p*	%	*p*	%	*p*	*p*	%	*p*	%	*p*	%
North(Beijing)	33.75	<0.001	25.45	<0.001	25.69	<0.001	21.58	<0.001	45.72	<0.001	54.36	<0.001	31.63	<0.001	27.66	<0.001	32.18	<0.001	22.38	<0.001	16.44	<0.001
South(Guangdong)	37.33		23.54		23.17		20.86		45.18		42.85		26.73		23.87		22.24		15.93		13.70	
East(Jiangsu, Shandong)	42.11		37.71		33.24		28.68		47.47		53.72		35.86		33.30		33.42		25.21		21.25	
West(Gansu, Yunnan)	42.63		35.70		28.60		26.29		48.09		46.13		28.19		26.99		27.80		22.11		20.70	
Central(Hubei)	33.14		25.94		20.70		22.26		39.70		43.65		24.25		25.66		21.26		14.95		15.02	
